# Rapid automated diagnosis of primary hepatic tumour by mass spectrometry and artificial intelligence

**DOI:** 10.1111/liv.14604

**Published:** 2020-08-04

**Authors:** Silvia Giordano, Sen Takeda, Matteo Donadon, Hidekazu Saiki, Laura Brunelli, Roberta Pastorelli, Matteo Cimino, Cristiana Soldani, Barbara Franceschini, Luca Di Tommaso, Ana Lleo, Kentaro Yoshimura, Hiroki Nakajima, Guido Torzilli, Enrico Davoli

**Affiliations:** ^1^ Mass Spectrometry Laboratory Environmental Health Sciences Department Istituto di Ricerche Farmacologiche Mario Negri IRCCS Milan Italy; ^2^ Department of Anatomy and Cell Biology University of Yamanashi Faculty of Medicine Chuo Japan; ^3^ Department of Hepatobiliary and General Surgery Humanitas University Humanitas Clinical and Research Center – IRCCS Milan Italy; ^4^ Laboratory of Hepatobiliary Immunopathology Humanitas Clinical and Research Center – IRCCS Milan Italy; ^5^ Shimadzu Corporation Kyoto Japan; ^6^ Department of Pathology Humanitas University Humanitas Clinical and Research Center – IRCCS Milan Italy; ^7^ Department of Internal Medicine Humanitas University Humanitas Clinical and Research Center – IRCCS Milan Italy; ^8^ Present address: Shimadzu Italia Srl Milan Italy

**Keywords:** artificial intelligence, liver cancer, liver surgery, liver tumours, mass spectrometry, resection margins

## Abstract

**Background and aims:**

Complete surgical resection with negative margin is one of the pillars in treatment of liver tumours. However, current techniques for intra‐operative assessment of tumour resection margins are time‐consuming and empirical. Mass spectrometry (MS) combined with artificial intelligence (AI) is useful for classifying tissues and provides valuable prognostic information. The aim of this study was to develop a MS‐based system for rapid and objective liver cancer identification and classification.

**Methods:**

A large dataset derived from 222 patients with hepatocellular carcinoma (HCC, 117 tumours and 105 non‐tumours) and 96 patients with mass‐forming cholangiocarcinoma (MFCCC, 50 tumours and 46 non‐tumours) were analysed by Probe Electrospray Ionization (PESI) MS. AI by means of support vector machine (SVM) and random forest (RF) algorithms was employed. For each classifier, sensitivity, specificity and accuracy were calculated.

**Results:**

The overall diagnostic accuracy exceeded 94% in both the AI algorithms. For identification of HCC vs non‐tumour tissue, RF was the best, with 98.2% accuracy, 97.4% sensitivity and 99% specificity. For MFCCC vs non‐tumour tissue, both algorithms gave 99.0% accuracy, 98% sensitivity and 100% specificity.

**Conclusions:**

The herein reported MS‐based system, combined with AI, permits liver cancer identification with high accuracy. Its bench‐top size, minimal sample preparation and short working time are the main advantages. From diagnostics to therapeutics, it has the potential to influence the decision‐making process in real‐time with the ultimate aim of improving cancer patient cure.

**Abbreviations**
AI: artificial intelligence
H&E: haematoxylin and eosin
HBV: hepatitis B virus
HCC: hepatocellular carcinoma
HCV: hepatitis C virus
*m*/*z*: mass to charge ratio
MFCC: mass‐forming cholangiocarcinoma
MS: mass spectrometry
NAFLD: non‐alcoholic fatty liver disease
NASH: non‐alcoholic steatohepatitis
PESI: probe electrospray ionization
PLS‐DA: partial least squares discriminant analysis
PPG: polypropylene glycol
REIMS: rapid evaporative ionization mass spectrometry
RF: random forest
RFA: radiofrequency ablation
SVM: support vector machine

**Key points**

We applied a mass spectrometry and artificial intelligence‐based approach to primary liver cancer rapid identification and classification. Once inter‐laboratory confirmatory studies are conducted, the proposed methodology might be translated into clinical practice with a wide range of applications in surgical oncology. From diagnostics to therapeutics, it has the potential to influence the decision‐making process in real‐time with the ultimate aim of improving cancer patient cure.

## 1 INTRODUCTION

Liver cancer, including hepatocellular carcinoma (HCC) and mass‐forming cholangiocarcinoma (MFCCC), is the seventh most common cancer and the fourth leading cause of cancer death worldwide.^1^ Despite a decrease in overall liver cancer mortality in Southern Europe and Japan, it has risen in some Northern and Central European countries, as well as in the United States, South America and Australia.^2^, ^3^, ^4^ In some countries, HCC resulting from steatohepatitis, such as non‐alcoholic steatohepatitis (NASH) or non‐alcoholic fatty liver disease (NAFLD), has increased.

Local therapies, such as radiofrequency ablation (RFA), hepatic resection, percutaneous ablations and transarterial treatments, are applied with curative intent,^4^, ^5^ while liver transplantation is the first choice for a limited number of patients. Traditional surgical methods for intra‐operative tissue identification are based on frozen section pathology analysis. This procedure, which involves rapid freezing, cutting, staining and examination, is time‐consuming, laborious and more importantly lacks in specificity. Moreover, pathological diagnosis can be affected by artefacts introduced during sample preparation. Histological diagnosis is usually based on the morphological characteristics of tumour cells and tissues which vary from patient to patient and even in the same nodule of a single liver (the so called ‘nodule in nodule’). Many different techniques have been applied in clinical settings: conventional morphological examination with Haematoxylin and Eosin (H&E) staining, immunohistochemistry and molecular biology.^6^ However, these procedures take 20‐30 minutes, so a more reliable, time‐efficient and less operator‐dependent technique is needed.

While conventional tumour diagnosis is based on morphological changes of cells and tissues, the chemical composition of tissue is also important to grasp the full profile of a particular tissue cell type. Mass spectrometry (MS) was introduced to the clinical field over 50 years ago and is commonly used to identify and quantify exogenous or endogenous molecules, like drugs, metabolites or proteins in tissue samples and blood by measuring the mass‐to‐charge ratio (*m/z*) of the molecular ions or their charged fragments.^7^, ^8^ For example, MS has been used as a powerful tool in screening for newborn congenital metabolic diseases.^9^ The MS profile can be used for classifying tissues and provides valuable prognostic information, such as tumour subtype and grade. Moreover, in recent years, MS technologies for tissue analysis have given promising results in refining the diagnosis and intra‐operative evaluation of surgical margins in common cancers, such as breast and pancreatic cancers, glioma, lung cancer, brain tumours and HCC.^10^, ^11^, ^12^, ^13^, ^14^, ^15^, ^16^, ^17^ These molecular‐based approaches are mainly based on the identification of MS signals specific to the tumour vs non‐tumour tissue or typical of a specific tumour subtype. The alterations identified are usually attributed to the modification of cellular metabolism or the tumour microenvironment.

Recently, ambient probe electrospray ionization (PESI) MS has been applied for the diagnosis of human renal cell carcinoma and chemically induced murine HCC.^18^, ^19^ The great advantage is the ability to generate ions directly from the tissues in real‐time, with minimal sample pretreatment.^20^ The operational ease of use and the real‐time assessment of tissue molecular information make MS extremely appealing, potentially meeting the requirements for routine clinical use.

With the aim to apply this novel methodology on liver cancer patients, we herein analysed a large dataset of Italian cancer patients using PESI‐MS instrumentation. Mass spectra data from these samples were used to build new artificial intelligence (AI) data algorithms. The system was validated in terms of concordance with a pathologist to determine whether it could usefully assist the clinicians in the clinical setting.

## 2 METHODS

### 2.1 Patients

The study was approved by the Ethics Committee of Humanitas University, Humanitas Clinical and Research Center – IRCCS (protocol ID 1705/2017). The tumour and the corresponding non‐tumour tissue samples were obtained from the institutional Cancer Center Bio‐bank and were selected by the pathologist of reference. Inclusion criteria for the selection of patients were the availability of written informed consent and the availability of clinical, oncological and pathological data. Patients whose data were missing were excluded. The samples in the certified bio‐bank were originally collected without compromising their diagnostic integrity, meaning that the total tissue was more than sufficient to achieve the final histological diagnosis. The following quality control procedures were used: the tumour and non‐tumour samples were thinly sliced by the pathologist and alternately submitted for histological assessment (paraffin‐embedded) or for biobanking in a mirror‐image fashion. The term ‘mirror‐image’ refers to embedding the frozen block so that its free, first‐cut surface corresponds to the cut surface of the immediately adjacent paraffin‐embedded block. Details of patients and tumours for all samples used in the study are shown in Tables S1 and S2.

### 2.2 Samples collection

Once the surgical piece was removed from a patient, the surgeon placed it in a sterile container and delivered it to the Department of Pathology at room temperature. The pathologist selected a fragment of representative tissue, carefully excluding any necrotic region, and the corresponding portion of non‐tumour tissue that was equally preserved. The selected tissue sample was divided into 50‐100 mg (~0.5 cm^3^) portions, and immediately frozen in liquid nitrogen and stored at −80°C until analysis.

### 2.3 Chemicals and reagents for MS

Triol‐type polypropylene glycol (PPGT, MW 300, 700, 1500) standard solutions were obtained from Wako Chemicals and the calibration solution was prepared in 2‐propanol 50%, NaCl 5%. Ethanol and 2‐propanol were purchased from Carlo Erba (Cornaredo) at chromatographic grade. Water was purified using a Milli‐Q system (Millipore).

### 2.4 PESI‐MS

Analysis was performed on a DPiMS‐2020™ (Shimadzu Corp.) based on the PESI ion source coupled to a single quadrupole mass analyzer. Instrument performance was verified at the start of each day of analysis, checking mass values and peak intensity ratios of a PPG mixture. For each sample, a 2 mm diameter piece of tissue was cut with a scalpel and homogenized with a PTFE pestle in 100 µL of ethanol/water (50:50). Ten microliter of the homogenized solution was dispensed in the solvent drip position of the sample plate and analysed. Analyses were performed in positive ion mode acquiring in full‐scan mode in the range of *m/z* 10‐2000. Mass spectra were acquired in full‐scan continuum mode (peak profile) and tables containing all mass peaks information were exported using LabSolutions software (Shimadzu Corp). After that, the exported *m/z* peak list was subjected to peak alignment (*m/z* tolerance 0.5 Da) and normalization on the TIC using eMSTAT Solution (Shimadzu Corp). The software eMSTAT was also used for multivariate partial least squares discriminant analysis (PLS‐DA). MS parameters are shown in Table 1. Each tissue sample was analysed for 2 minutes in order to have a number of mass spectra sufficient to maximize signal‐to‐noise ratio and to maintain optimal ionization process, preventing loss of signal caused by PESI needle contamination.

**TABLE 1:** Mass spectrometry parameters for hepatocellular carcinoma and mass‐forming cholangiocarcinoma analyses

Polarity	Positive
Mass range	*m/z* 10‐2000
Desolvation line	250°C
Heat block	50°C
Nebulizing gas	0 L/min
Drying gas	0 L/min

### 2.5 Application of artificial intelligence

Each 2‐minute sample acquisition was arbitrary divided into fragments of 10 seconds each. Mass spectra were averaged for every acquisition fragment and 12 mass spectra were exported for analysis. The last two fragments were discarded in order to give a more consistent signal intensity. Thus, for each sample, 10 acquisition fragments were used for machine learning. As a first step, we tested the ability of the system to distinguish HCC and MFCCC from the respective non‐tumour tissues. In the second step, we tested its ability to classify HCC separately from MFCCC, comparing one tumour group against the other one. Two different algorithms, support vector machine (SVM) and random forest (RF), were tested for machine learning and classification, being both available with eMSTAT Solution software (Shimadzu Corp.). Both models were evaluated using the *K*‐Fold cross‐validation method (*K* = 10). The accuracy of these statistical models was evaluated in terms of concordance with pathologist classification. Statistical validation and evaluation of the SVM and RF models were done using the leave‐many‐out cross‐validation method: for each classification model, all of the collected fragments were split into 10 sets and each set was then tested against the others used for machine learning. To prevent data leakage, data fragments from the same patient and sample were included in the same cross‐validation dataset.

## 3 RESULTS

### 3.1 Patients' clinical characteristics

According to the aforementioned selection criteria, the study comprised a consecutive cohort of 117 HCC and 50 MFCCC patients for a total of 167 patients, who underwent liver resection in the Department of Hepatobiliary and General Surgery of Humanitas University, Humanitas Clinical and Research Center in Milan (Italy) from 1 June 2011 to 31 December 2018. Among HCC patients, we collected 117 tumour and 105 non‐tumour samples while for MFCCC, we collected 50 tumour and 46 non‐tumour samples.

Of the HCC patients, 95 (82%) were men and 22 (18%) were women. The median age of cohort was 71 years (ranging from 27 to 85). Most of the patients (91%) had underlying chronic hepatitis or cirrhosis, which was associated with HCV, HBV or alcohol in 28%, 16% and 19% of the patients respectively. Sixty‐two patients (53%) had microvascular invasion, while 14 (12%) also had macrovascular invasion. Among the MFCCC patients, 37 (74%) were men and 13 (26%) were women. The median age of cohort was 67 years (ranging from 31 to 74). Most of the patients (78%) had an underlying normal liver with no known risk factors for developing such tumours. Thirty‐five patients (68%) had vascular invasion.

### 3.2 PESI‐MS analysis

**FIGURE 1:**
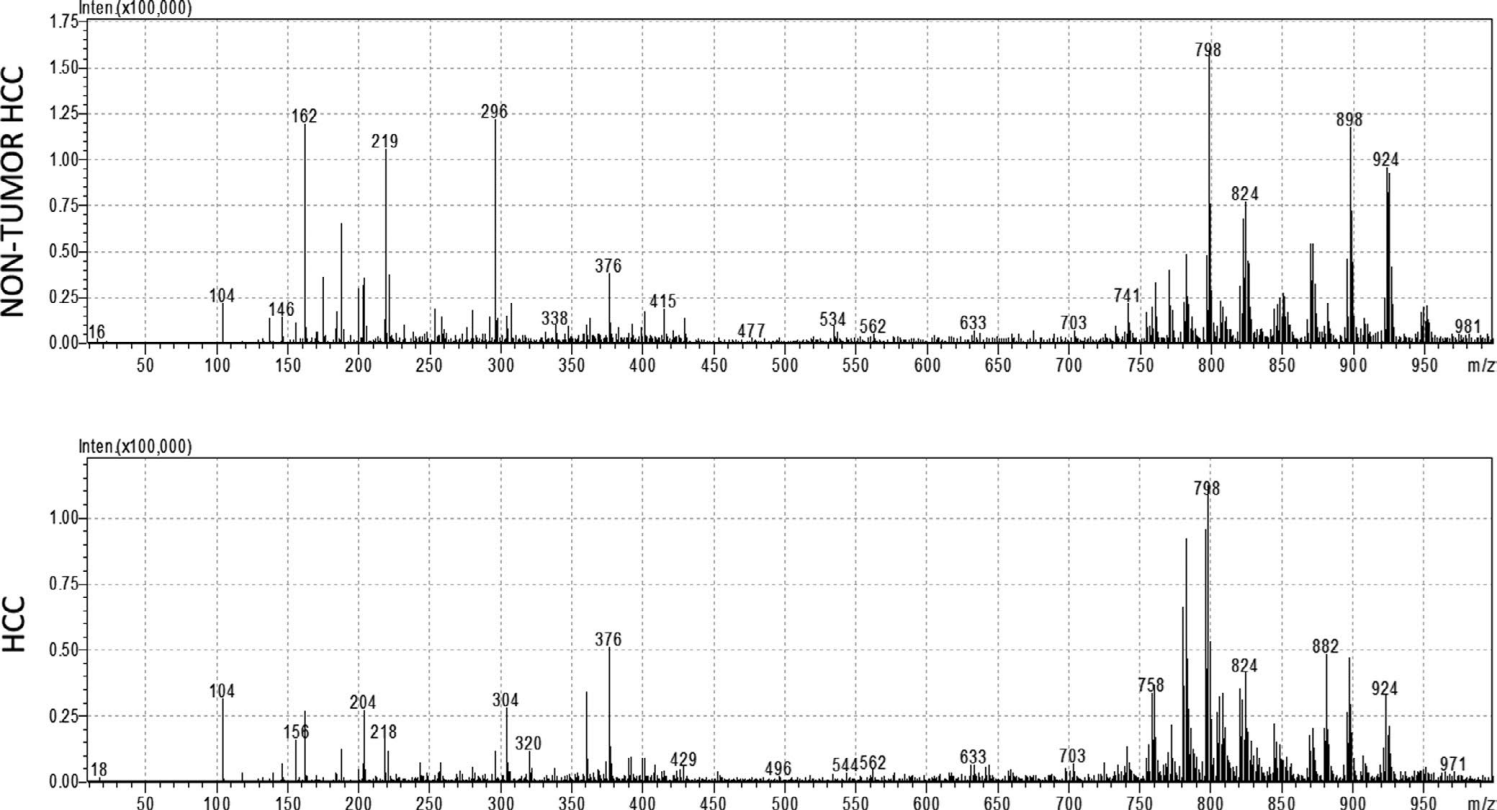
Representative mass spectra of non‐tumour liver tissue (upper panel) and hepatocellular carcinoma (HCC) (lower panel) from the same patient. Each spectrum is the average of a 10‐second acquisition fragment

Figure 1 shows two representative full‐scan mass spectra, averaged from 10‐second acquisition fragments, from the tumour‐free part of the liver and the tumour counterpart from the same patient.

**FIGURE 2:**
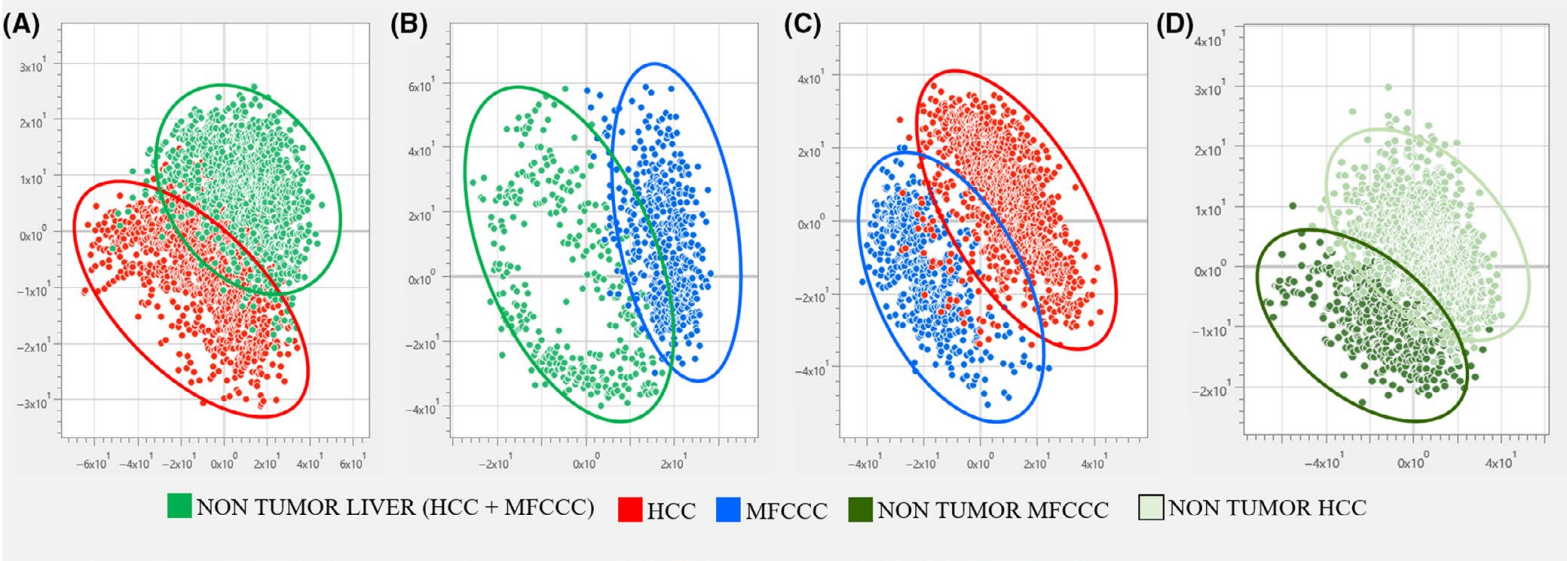
Partial least squares discriminant analysis score plot of (A) hepatocellular carcinoma (HCC) vs non‐tumour liver tissue; (B) mass‐forming cholangiocarcinoma (MFCCC) vs non‐tumour liver tissue; (C) HCC vs CCC; and (D) non‐tumour HCC vs non‐tumour MFCCC

For each sample, 10 spectral fragments were extracted from the total ion chromatogram to meet the selection criteria, for a grand total of 3180. We applied multivariate statistical analysis to test whether these datasets were separate from each other (Figure 2). PLS‐DA showed good separation of HCC from tumour‐free tissues (Figure 2A), and MFCCC from tumour‐free tissues (Figure 2B), while some overlapping dots from both groups still remained. PLS‐DA also distinguished HCC from MFCCC (Figure 2C) but not the non‐tumour samples of HCC patients vs those of MFCCC patients (Figure 2D).

**FIGURE 3:**
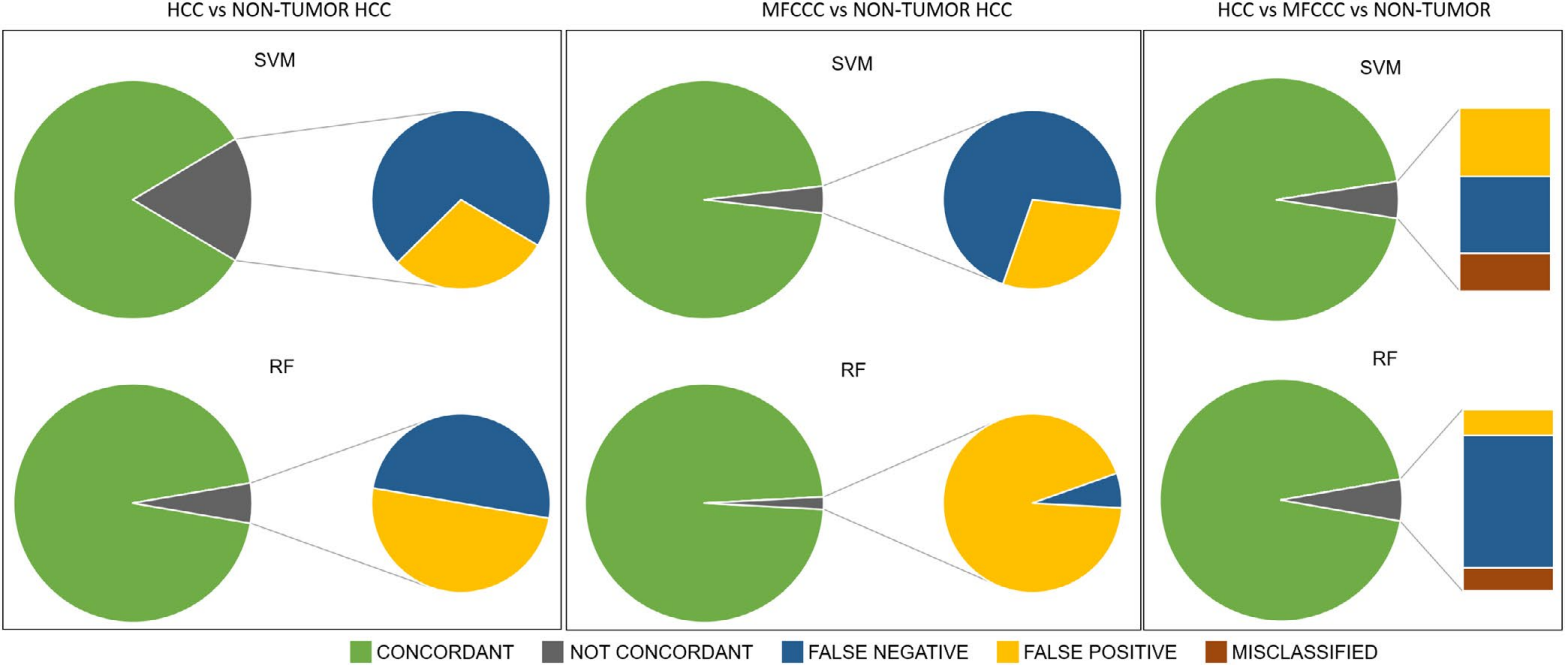
Support vector machine (SVM) and random forest (RF) machine learning algorithm results on mass spectra fragments. Green= Concordant, Grey=Not corcordant, Blue=False negative, Yellow=False positive, Red=Misclassified

Two kinds of machine learning algorithm were then applied, SVM and RF, to distinguish the following combinations: HCC vs tumour‐free tissues, MFCCC vs tumour‐free tissues and HCC vs MFCCC vs tumour‐free tissues. We tested the accuracy of each model by the leave‐one‐out cross‐validation method: technically, all groups were split into 10 separate sets and each set was tested against the others. The results for each algorithm are reported in Tables S3‐S5 and summarized in Figure 3. For HCC vs tumour‐free tissues, 2220 acquisition spectral fragments were used for validation. Concordance with the pathological diagnosis was 83.2% for SVM, with 262 extracted fragments resulting false positive and 110 false negative of 2220 (12% and 5% respectively). In this case, sensitivity was 77.6% and specificity was 89.5%. RF gave higher concordance than SVM, with 2100 fragments correctly assigned (94.6%): non‐concordant cases included 60 false negatives and 60 false positives, with a sensitivity of 94.9% and a specificity of 94.3% (Figure 3A; Table S3).

For MFCCC vs tumour‐free tissue, 925 of 960 fragments used for testing the SVM algorithm were correctly assigned (96.4%). Of the remaining 35 fragments, 10 were false positive (specificity 97.8%) and 25 were false negative (sensitivity 95%). The concordance rate with pathological diagnosis for the RF algorithm was 98.3%, with a sensitivity of 99.8% and a specificity of 96.7% (Figure 3B; Table S4).

We applied this system to classify the histological types of liver cancer, distinguishing HCC from MFCCC. The accuracy of SVM was 95.1%: among the non‐concordant fragments, 37.1% were false positive, 42.3% were false negative and 20.6% were misclassified, that is mass spectra from HCC classified as MFCCC or vice versa. For RF, the accuracy was 94%, with 120 fragments not showing concordance with the pathological diagnosis; 14 of these (11.7%) were misclassified (Figure 3C; Table S5).

The final PESI‐MS system judgement is based on the majority decisions on the 10 fragments extracted. Technically, if five or more acquisition fragments in a single specimen are classified correctly, the sample is judged as concordant; if fewer than five, the judgement is non‐concordant. This approach gave a higher concordance rate than the previous one (Tables S6‐S8), identifying HCC separately from tumour‐free tissue reached 88.7% in SVM (25 of 222 specimens were discordant) and 98.2% in RF (with only four samples not concordant). For the diagnosis of MFCCC from tumour‐free tissue, the concordance rate was 99.0% for both algorithms, with only one false negative of 96 cases. The classification of tumour types reached 98.5% concordance in SVM, where three cases were misclassified (one false positive, one false negative and one HCC classified as MFCCC), and 94.9% in the RF algorithm, with 10 misclassified cases.

## 4 CONCLUSIONS

Complete surgical resection with negative margins remains an important pillar in surgery for liver tumours. And accurate intra‐operative evaluation of residual tumours on the resection margin or classification of small new nodules of uncertain origin found during surgery is the critical step to improve overall patient survival. Currently, the presence of cancer cells in tumour margins is scanned intra‐operatively by ultrasonography with reliable results^21^ or less frequently by rapid frozen pathology. However, several limitations still remain and we have to cope with those by alternatives.

The MS techniques that have been successfully used to characterize tumour resection margins are currently grouped into two main categories: MS imaging (MSI) of tissue slices, and direct tissue sampling under ambient conditions.^22^, ^23^, ^24^ MSI can be very useful for tissue classification based on the distribution of specific molecules, but its translation into routine clinical and surgical setting is complex. Moreover, because of its time‐consuming sample preparation, MSI does not overcome the conventional immunocytochemical staining. Since its introduction into the operating room (OR) over a decade ago,^25^ a number of MS techniques have been developed and classified into two main groups: online direct intra‐operative MS and offline sampling probe‐based methods. The online techniques employ classical tissue manipulation methods that, during surgery, cause the thermal or mechanical disintegration of tissues, producing an aerosol of charged droplets which can yield gas phase ions. The aerosol is sampled with a vacuum pump and delivered to the mass spectrometer inlet orifice. Two of the most commonly used online approaches are Rapid Evaporative Ionization Mass Spectrometry (REIMS)^13^, ^26^, ^27^ and the MasSpec Pen.^17^, ^28^ All data points in REIMS are acquired and analysed within 3 seconds, with specificity that ranges from 92% to 100% for positive tissue identification.^29^ Offline methods require a small tissue portion to be sampled and transported to the MS where it is analysed. While the online solutions last a few seconds but are isolated from the pathology, offline approaches take longer but are integrated with the pathology.

In this study, we developed a new automated system to identify and classify, with high diagnostic accuracy, liver cancer tissue from a large dataset of patients. While both positive and negative MS ionization modes were tested in developing the system, only positive ion mode gave valuable data being capable of discriminating the cancer from non‐can

cer specimens (data not shown). This could be the consequence of an alteration, during cancer progression, to a larger number of molecular species that dominate the mass spectrum in positive ion mode.^30^ Studies are ongoing to address this point, with metabolomics approaches, in order to identify molecular species, mainly involved in this process. Ninety‐six MFCCC and 222 HCC were collected and analysed by our MS instrument combined with a dedicated AI calculation model. The mass spectra of 10 fragments, averaged from a 10‐second acquisition for each analysis, were exported to the database; a total of 3180 mass spectra were used for statistical analysis in order to minimize errors caused by instrumental noise and ionization variability. The PLS‐DA analysis showed a good separation of non‐tumour samples vs tumour samples (Figure 2A,B) and between the two different tumour histotypes (Figure 2C) showing the ability of the system to distinguish these samples. As reported in Figure 2D, the non‐tumour samples were not pooled but each patient had their own non‐tumour control sample, taken and assessed by the hepatobiliary pathologist. Comparing non‐tumour samples of HCC patients vs those of MFCCC patients, there is a minor separation using PLS‐DA. From the biological and clinical standpoints, it is reasonable that these two non‐tumour samples were not completely overlapped: the non‐tumoural samples were free of cancer cells but they might differ in the amount of steatosis, fibrosis and inflammation. However, such diversity of non‐tumour tissue was not the object of this research and might be further investigated in the next future.

Although there were reports of differences in molecular patterns between tumour and tumour‐free liver tissues,^10^, ^27^, ^28^ they chiefly focus on the identification of each signal alteration in a specific tissue type.^31^ In our study, we made the most of whole spectra without selecting specific peaks (Figure 2). By appropriately augmenting and controlling the size of database, this system gains more accuracy in prediction power. As our initial aim is to make binary judgement of tumour and non‐tumour, we decided to employ renowned machine learning algorithm, such as SVM and RF.^32^, ^33^ These models, compared to deep learning algorithms like artificial neural networks, can be implemented with relatively smaller dataset, being probably better suited for clinical samples, and are easier and faster to train. Their predictive accuracy was validated by the *K*‐fold cross‐validation method (*K* = 10), that is all mass spectra acquired were split into 10 sets and each set was tested against the others used for training. We took care not to include the mass spectra from the same analysis into the simultaneous set used for training and testing, to avoid over‐fitting of algorithm. Under this condition, the power of prediction was 95% in all cases. For the classification of HCC and tumour‐free tissue class, RF was the best with 98.2% accuracy; for MFCCC vs tumour‐free tissue, accuracy was 99% in both SVM and RF, with only one misclassified sample. This means that only one patient of 96 was misdiagnosed by both algorithms.

While MFCCC accounts for only 5% of primary liver malignancies, it is often difficult to make a differential diagnosis from HCC, and a definitive conclusion is vital to choose appropriate therapeutics. Our multivariate statistical analysis showed the possibility of the system to distinguish tumour‐free tissues from both HCC and MFCCC, with limited overlap causing false positive or negative outcomes. Moreover, PLS‐DA can distinguish the two tumour types. This is pivotal as each histotype may require a different therapeutic strategy. Indeed, even though these two tumours usually have different radiological features that are used for differential diagnosis, several other factors can complicate their clinical management.^34^ An accurate and valid method to distinguish HCC from MFCCC on the basis of the physicochemical parameters of each tumour would be of paramount importance in clinical settings – especially when only an ambiguous decision is possible from the radiological findings. Of note, this last result, which is the capability of an automated system to distinguish between two different tumour histotypes, has never been reported and open important applications in the burgeoning field of AI in medicine and surgery.

Importantly, the approach used in this study requires minimal sample pretreatment with almost real‐time analysis, making it possible to determine tumour resection margins in surgical practice. Several approaches are now showing increasingly more frequently how MS, combined with AI, is a reliable emerging technique for tissue classification. This is possible using different instrumental technologies, with different levels of complexity. The high accuracy of the results presented here, obtained with entry‐level equipment, demonstrates that this approach might be transferred into clinical practice, once inter‐laboratory confirmatory studies are conducted. PESI‐MS is a bench‐top, fully automated system that can be used by personnel with limited analytical training. Its positioning in the OR cuts down the steps needed for conventional pathological diagnosis, which risk introducing artefacts. Intra‐operative pathological examination of frozen sections is often time‐consuming and sometimes unreliable. An objective MS‐based approach for direct tissue analysis may help to tackle some of the challenges still encountered in daily clinical practice, including the difficulty in providing a detailed histological definition, inter‐observer variance across centres and a delay in diagnosis. A clinical trial on this MS‐based technology is already on course in Japan. Preliminary data are promising and will be object of our future publication.

The main limitation of this study is that all samples came from the same clinical centre. This could limit the machine learning ability to cope with inter‐institutional differences in specimens. However, even considering that the results reported need to be confirmed in other centres, this study provides compelling evidence that the proposed methodology can be translated into clinical practice with a wide range of applications in surgical oncology. From diagnostics to therapeutics, it has the potential to influence the decision‐making process in real‐time with the ultimate aim of improving cancer patient cure.

## CONFLICT OF INTEREST

The authors declare that there are no conflict of interest regarding the publication of this paper.

## Ethics approval statement

The study was approved by the Ethics Committee of Humanitas University, Humanitas Clinical and Research Center – IRCCS (protocol ID 1705/2017).

## Supporting information

Tables S1‐S8
