# Supplementary material for: Rapid automated diagnosis of primary hepatic tumour by mass spectrometry and artificial intelligence
Source: Liver Int. 2020 Aug 4;40(12):3117–24. doi: 10.1111/liv.14604 (PMC7754124; doi:10.1111/liv.14604)
Supplement: Supplementary file 1 — Tables S1‐S8 [file LIV-40-3117-s001.docx]

**Rapid automated diagnosis of primary hepatic tumor by mass spectrometry and artificial intelligence**

Silvia Giordano^1† ‡^, Sen Takeda^2†^, Matteo Donadon^3,4^, Hidekazu Saiki^5^, Laura Brunelli^1^, Roberta Pastorelli^1^, Matteo Cimino^3,4^, Cristiana Soldani^3^, Barbara Franceschini^3^, Luca Di Tommaso^6^, Ana Lleo^4,7^, Kentaro Yoshimura^2^, Hiroki Nakajima^5^, Guido Torzilli^3,4^ and Enrico Davoli^1§^

**Affiliations**

1. Mass Spectrometry Laboratory, Environmental Health Sciences Department, Istituto di Ricerche Farmacologiche Mario Negri IRCCS, Via Mario Negri 2, 20156 Milan, Italy
2. Department of Anatomy and Cell Biology, University of Yamanashi Faculty of Medicine, 1110 Shimo-Kateau, Chuo, Yamanashi, Japan
3. Department of Hepatobiliary and General Surgery, Humanitas University, Humanitas Clinical and Research Center – IRCCS, Via Manzoni 56, Rozzano, Milan, Italy
4. Laboratory of Hepatobiliary Immunopathology, Humanitas Clinical and Research Center – IRCCS, Via Manzoni 56, Rozzano, Milan, Italy
5. Shimadzu Corporation, 1, Nishinokyo-Kuwabara-cho, Nakagyo-ku, Kyoto 604-8511, Japan
6. Department of Pathology, Humanitas University, Humanitas Clinical and Research Center – IRCCS, Via Manzoni 56, Rozzano, Milan, Italy
7. Department of Internal Medicine, Humanitas University, Humanitas Clinical and Research Center – IRCCS, Via Manzoni 56, Rozzano, Milan, Italy

^†^ These authors contributed equally to this work.

^‡^ Actual address Shimadzu Italia Srl, Via Giovanni Battista Cassinis, 7, 20139 Milano MI

^§^ Corresponding author

**Table S1:** Main details of HCC patients (N=117)

|  | **N (%)** |
| --- | --- |
| **Demographic** |  |
| *Age (year)* |  |
| Median; range | 71; 27-85 |
| >65 years | 86 (74) |
| *Sex* |  |
| M/F | 95 (81) / 22 (19) |
| **Underlying liver** |  |
| Etiology |  |
| HCV | 33 (28) |
| HBV | 19 (16) |
| Alcohol | 22 (19) |
| Unknown | 43 (37) |
| Quality of the liver |  |
| Chronic hepatitis or cirrhosis | 106 (91) |
| Normal liver | 11 (9) |
| Child-Pugh-Turcotte score A | 117 (100) |
| Model for End-stage Liver Disease |  |
| Median; range | 8; 6-16 |
| **Tumor details** |  |
| Tumor size (cm) |  |
| Median; range | 5.3; 1.4-28 |
| Tumor number |  |
| Total resected tumor | 129 |
| Median; range | 1; 1-12 |
| Barcelona Clinic Liver Cancer stage |  |
| 0-A | 61 (52) |
| B | 42 (36) |
| C | 14 (12) |
| Alpha Fetoprotein |  |
| Median; range | 6.1; 1-29.011 |
| Vascular Invasion |  |
| Micro | 62 (53) |
| Macro | 14 (12) |
| Grading |  |
| 1-2 | 79 (67) |
| 3-4 | 38 (33) |
| **Surgical details** |  |
| *Extent of hepatectomy* |  |
| Major (>3 segments) | 15 (13) |
| Minor | 102 (87) |
| *Type of hepatectomy* |  |
| Anatomical | 51 (44) |
| Non-anatomical | 66 (56) |

**Table S2:** Main details of MFCCC patients (N=50)

|  | **N (%)** |
| --- | --- |
| **Demographic** |  |
| Age (year) |  |
| Median; range | 67; 31-74 |
| >65 years | 35 (70) |
| Sex |  |
| M/F | 37 (74) / 13 (26) |
| **Underlying liver** |  |
| Etiology |  |
| HCV | 12 (24) |
| HBV | 2 (4) |
| Alcohol | 4 (8) |
| Unknown | 14 (28) |
| Quality of the liver |  |
| Chronic hepatitis or cirrhosis | 11 (22) |
| Normal liver | 39 (78) |
| Child-Pugh-Turcotte score A | 50 (100) |
| Model for End-stage Liver Disease |  |
| Median; range | 7; 5-11 |
| **Tumor details** |  |
| Tumor size (cm) |  |
| Median; range | 3.9; 1.9-16 |
| Tumor number |  |
| Total resected tumor | 63 |
| Median; range | 1; 1-13 |
| Serum level of Ca 19-9 |  |
| Median; range | 31.1; 23.1-1458 |
| Vascular Invasion |  |
| Micro | 23 (46) |
| Macro | 12 (24) |
| Grading |  |
| 1-2 | 18 (36) |
| 3-4 | 32 (64) |
| **Surgical details** |  |
| Extent of hepatectomy |  |
| Major (>3 segments) | 18 (36) |
| Minor | 32 (64) |
| Type of hepatectomy |  |
| Anatomical | 21 (42) |
| Non-anatomical | 29 (58) |

**Table S3:** Machine learning results for HCC acquisition fragments.

**SUPPORT VECTOR MACHINE**

|  | **HCC** | | | | **NON-TUMOR TISSUE** | | | | **TOTAL** | | | |
| --- | --- | --- | --- | --- | --- | --- | --- | --- | --- | --- | --- | --- |
|  | **TRAINING** | **TEST** | **CORRECT** | **SENSITIVITY (%)** | **TRAINING** | **TEST** | **CORRECT** | **SPECIFICITY (%)** | **TRAINING** | **TEST** | **CORRECT** | **ACCURACY (%)** |
| **SET 1** | 1050 | 120 | 48 | 40 | 940 | 110 | 110 | 100 | 1990 | 230 | 158 | 68.7 |
| **SET 2** | 1060 | 110 | 94 | 85.5 | 940 | 110 | 102 | 92.7 | 2000 | 220 | 196 | 89.1 |
| **SET 3** | 1060 | 110 | 104 | 94.5 | 950 | 100 | 87 | 87.0 | 2010 | 210 | 191 | 91.0 |
| **SET 4** | 1050 | 120 | 48 | 40.0 | 930 | 120 | 101 | 84.2 | 1980 | 240 | 149 | 62.1 |
| **SET 5** | 1050 | 120 | 117 | 97.5 | 950 | 100 | 92 | 92 | 2000 | 220 | 209 | 95.0 |
| **SET 6** | 1040 | 130 | 110 | 84.6 | 940 | 110 | 86 | 78.2 | 1980 | 240 | 196 | 81.7 |
| **SET 7** | 1050 | 120 | 105 | 87.5 | 950 | 100 | 93 | 93.0 | 2000 | 220 | 198 | 90.0 |
| **SET 8** | 1050 | 120 | 77 | 64.2 | 930 | 120 | 112 | 93.3 | 1980 | 240 | 189 | 78.8 |
| **SET 9** | 1050 | 120 | 119 | 99.2 | 930 | 120 | 113 | 94.2 | 1980 | 240 | 232 | 96.7 |
| **SET 10** | 1070 | 100 | 86 | 86.0 | 990 | 60 | 44 | 73.3 | 2060 | 160 | 130 | 81.3 |
| **TOTAL** | 10530 | 1170 | 908 | 77.6 | 9450 | 1050 | 940 | 89.5 | 19980 | 2220 | 1848 | **83**.**2** |

**RANDOM FOREST**

|  | **HCC** | | | | **NON-TUMOR TISSUE** | | | | **TOTAL** | | | |
| --- | --- | --- | --- | --- | --- | --- | --- | --- | --- | --- | --- | --- |
|  | **TRAINING** | **TEST** | **CORRECT** | **SENSITIVITY (%)** | **TRAINING** | **TEST** | **CORRECT** | **SPECIFICITY (%)** | **TRAINING** | **TEST** | **CORRECT** | **ACCURACY (%)** |
| **SET 1** | 1050 | 120 | 114 | 95 | 940 | 110 | 99 | 90 | 1990 | 230 | 213 | 92.6 |
| **SET 2** | 1060 | 110 | 97 | 88.2 | 940 | 110 | 102 | 92.7 | 2000 | 220 | 199 | 90.5 |
| **SET 3** | 1060 | 110 | 107 | 97.3 | 950 | 100 | 90 | 90 | 2010 | 210 | 197 | 93.8 |
| **SET 4** | 1050 | 120 | 119 | 99.2 | 930 | 120 | 118 | 98.3 | 1980 | 240 | 237 | 98.8 |
| **SET 5** | 1050 | 120 | 120 | 100 | 950 | 100 | 90 | 90 | 2000 | 220 | 210 | 95.5 |
| **SET 6** | 1040 | 130 | 128 | 98.5 | 940 | 110 | 106 | 96.4 | 1980 | 240 | 234 | 97.5 |
| **SET 7** | 1050 | 120 | 110 | 91.7 | 950 | 100 | 94 | 94 | 2000 | 220 | 204 | 92.7 |
| **SET 8** | 1050 | 120 | 108 | 90 | 930 | 120 | 120 | 100 | 1980 | 240 | 228 | 95 |
| **SET 9** | 1050 | 120 | 120 | 100 | 930 | 120 | 116 | 96.7 | 1980 | 240 | 236 | 98.3 |
| **SET 10** | 1070 | 100 | 87 | 87.0 | 990 | 60 | 55 | 91.7 | 2060 | 160 | 142 | 88.8 |
| **TOTAL** | 10530 | 1170 | 1110 | 94.9 | 9450 | 1050 | 990 | 94.3 | 19980 | 2220 | 2100 | **94**.**6** |

**Table S4:** Machine learning results for MFCCC acquisition fragments.

**SUPPORT VECTOR MACHINE**

|  | **MFCCC** | | | | **NON-TUMOR TISSUE** | | | | **TOTAL** | | | |
| --- | --- | --- | --- | --- | --- | --- | --- | --- | --- | --- | --- | --- |
|  | **TRAINING** | **TEST** | **CORRECT** | **SENSITIVITY (%)** | **TRAINING** | **TEST** | **CORRECT** | **SPECIFICITY (%)** | **TRAINING** | **TEST** | **CORRECT** | **ACCURACY (%)** |
| **SET 1** | 440 | 60 | 50 | 83.3 | 410 | 50 | 49 | 98 | 850 | 110 | 99 | 90 |
| **SET 2** | 410 | 50 | 50 | 100 | 450 | 50 | 48 | 96 | 860 | 100 | 98 | 98 |
| **SET 3** | 440 | 60 | 56 | 93.3 | 400 | 60 | 60 | 100 | 840 | 120 | 116 | 96.7 |
| **SET 4** | 440 | 60 | 58 | 96.7 | 410 | 50 | 50 | 100 | 850 | 110 | 108 | 98.2 |
| **SET 5** | 440 | 60 | 60 | 100 | 400 | 60 | 58 | 96.7 | 840 | 120 | 118 | 98.3 |
| **SET 6** | 440 | 60 | 54 | 90 | 410 | 50 | 50 | 100 | 850 | 110 | 104 | 94.5 |
| **SET 7** | 440 | 60 | 60 | 100 | 400 | 60 | 60 | 100 | 840 | 120 | 120 | 100 |
| **SET 8** | 450 | 50 | 50 | 100 | 410 | 50 | 45 | 90 | 860 | 100 | 95 | 95 |
| **SET 9** | 460 | 40 | 37 | 92.5 | 430 | 30 | 30 | 100 | 890 | 70 | 67 | 95.7 |
| **TOTAL** | 3960 | 500 | 475 | 95 | 3720 | 460 | 450 | 97.8 | 7680 | 960 | 925 | **96**.**4** |

**RANDOM FOREST**

|  | **MFCCC** | | | | **NON-TUMOR TISSUE** | | | | **TOTAL** | | | |
| --- | --- | --- | --- | --- | --- | --- | --- | --- | --- | --- | --- | --- |
|  | **TRAINING** | **TEST** | **CORRECT** | **SENSITIVITY (%)** | **TRAINING** | **TEST** | **CORRECT** | **SPECIFICITY (%)** | **TRAINING** | **TEST** | **CORRECT** | **ACCURACY (%)** |
| **SET 1** | 440 | 60 | 60 | 100 | 410 | 50 | 50 | 100 | 850 | 110 | 110 | 100 |
| **SET 2** | 410 | 50 | 50 | 100 | 450 | 50 | 48 | 96 | 860 | 100 | 98 | 98 |
| **SET 3** | 440 | 60 | 59 | 98.3 | 400 | 60 | 49 | 81.7 | 840 | 120 | 108 | 90 |
| **SET 4** | 440 | 60 | 60 | 100 | 410 | 50 | 49 | 98 | 850 | 110 | 109 | 99.1 |
| **SET 5** | 440 | 60 | 60 | 100 | 400 | 60 | 60 | 100 | 840 | 120 | 120 | 100 |
| **SET 6** | 440 | 60 | 60 | 100 | 410 | 50 | 50 | 100 | 850 | 110 | 110 | 100 |
| **SET 7** | 440 | 60 | 60 | 100 | 400 | 60 | 60 | 100 | 840 | 120 | 120 | 100 |
| **SET 8** | 450 | 50 | 50 | 100 | 410 | 50 | 49 | 98 | 860 | 100 | 99 | 99 |
| **SET 9** | 460 | 40 | 40 | 100 | 430 | 30 | 30 | 100 | 890 | 70 | 70 | 100 |
| **TOTAL** | 3960 | 500 | 499 | 99.8 | 3720 | 460 | 445 | 96.7 | 7680 | 960 | 944 | **98**.**3** |

**Table S5:** Machine learning results for HCC and MFCCC acquisition fragments.

**SUPPORT VECTOR MACHINE**

|  | **TUMOR** | | | | | | | | **NON-TUMOR TISSUE** | | | | | | | | **TOTAL** | | | |
| --- | --- | --- | --- | --- | --- | --- | --- | --- | --- | --- | --- | --- | --- | --- | --- | --- | --- | --- | --- | --- |
|  | **HCC** | | | | **MFCCC** | | | | **HCC** | | | | **MFCCC** | | | |  | | | |
|  | **TR** | **TE** | **COR** | **SENS. (%)** | **TR** | **TE** | **COR** | **SENS. (%)** | **TR** | **TEST** | **COR** | **SPEC. (%)** | **TR** | **TE** | **COR** | **SPEC. (%)** | **TR** | **TE** | **C** | **ACC (%)** |
| **SET 1** | 450 | 50 | 48 | 96 | 450 | 50 | 50 | 100 | 450 | 50 | 50 | 100 | 410 | 50 | 50 | 100 | 1760 | 200 | 198 | 99 |
| **SET 2** | 450 | 50 | 46 | 92 | 450 | 50 | 50 | 100 | 450 | 50 | 46 | 92 | 420 | 40 | 40 | 100 | 1770 | 190 | 182 | 95.8 |
| **SET 3** | 450 | 50 | 49 | 98 | 450 | 50 | 44 | 88 | 450 | 50 | 45 | 90 | 410 | 50 | 46 | 92 | 1760 | 200 | 184 | 92 |
| **SET 4** | 450 | 50 | 48 | 96 | 430 | 70 | 68 | 97.1 | 450 | 50 | 49 | 98 | 420 | 40 | 37 | 92.5 | 1750 | 210 | 202 | 96.2 |
| **SET 5** | 450 | 50 | 48 | 96 | 440 | 60 | 60 | 100 | 450 | 50 | 49 | 98 | 420 | 40 | 39 | 97.5 | 1760 | 200 | 196 | 98 |
| **SET 6** | 450 | 50 | 36 | 72 | 460 | 40 | 39 | 97.5 | 450 | 50 | 47 | 94 | 410 | 50 | 50 | 100 | 1770 | 190 | 172 | 90.5 |
| **SET 7** | 450 | 50 | 40 | 80 | 440 | 60 | 60 | 100 | 450 | 50 | 44 | 88 | 410 | 50 | 50 | 100 | 1750 | 210 | 194 | 92.4 |
| **SET 8** | 450 | 50 | 46 | 92 | 450 | 50 | 50 | 100 | 450 | 50 | 48 | 96 | 410 | 50 | 50 | 100 | 1760 | 200 | 194 | 97 |
| **SET 9** | 450 | 50 | 47 | 94 | 450 | 50 | 50 | 100 | 450 | 50 | 49 | 98 | 410 | 50 | 49 | 98 | 1760 | 200 | 195 | 97.5 |
| **SET 10** | 450 | 50 | 44 | 88 | 450 | 50 | 46 | 92 | 450 | 50 | 48 | 96 | 420 | 40 | 38 | 95 | 1770 | 190 | 176 | 92.6 |
| **TOTAL** | 4500 | 500 | 452 | 90.4 | 4470 | 530 | 517 | 97.5 | 4500 | 500 | 475 | 95 | 4140 | 460 | 449 | 97.6 | 17610 | 1990 | 1893 | **95**.**1** |

**RANDOM FOREST**

|  | **TUMOR** | | | | | | | | **NON-TUMOR TISSUE** | | | | | | | | **TOTAL** | | | |
| --- | --- | --- | --- | --- | --- | --- | --- | --- | --- | --- | --- | --- | --- | --- | --- | --- | --- | --- | --- | --- |
|  | **HCC** | | | | **MFCCC** | | | | **HCC** | | | | **MFCCC** | | | |  | | | |
|  | **TR** | **TE** | **COR** | **SENS. (%)** | **TR** | **TE** | **COR** | **SENS. (%)** | **TR** | **TEST** | **COR** | **SPEC. (%)** | **TR** | **TE** | **COR** | **SPEC. (%)** | **TR** | **TE** | **C** | **ACC (%)** |
| **SET 1** | 450 | 50 | 50 | 100 | 450 | 50 | 50 | 100 | 450 | 50 | 42 | 84 | 410 | 50 | 50 | 100 | 1760 | 200 | 192 | 96 |
| **SET 2** | 450 | 50 | 32 | 64 | 450 | 50 | 50 | 100 | 450 | 50 | 50 | 100 | 420 | 40 | 40 | 100 | 1770 | 190 | 172 | 90.5 |
| **SET 3** | 450 | 50 | 50 | 100 | 450 | 50 | 48 | 96 | 450 | 50 | 43 | 86 | 410 | 50 | 50 | 100 | 1760 | 200 | 191 | 95.5 |
| **SET 4** | 450 | 50 | 49 | 98 | 430 | 70 | 69 | 98.6 | 450 | 50 | 50 | 100 | 420 | 40 | 40 | 100 | 1750 | 210 | 208 | 99 |
| **SET 5** | 450 | 50 | 44 | 88 | 440 | 60 | 60 | 100 | 450 | 50 | 50 | 100 | 420 | 40 | 40 | 100 | 1760 | 200 | 194 | 97 |
| **SET 6** | 450 | 50 | 34 | 68 | 460 | 40 | 30 | 75 | 450 | 50 | 50 | 100 | 410 | 50 | 50 | 100 | 1770 | 190 | 164 | 86.3 |
| **SET 7** | 450 | 50 | 34 | 68 | 440 | 60 | 60 | 100 | 450 | 50 | 49 | 98 | 410 | 50 | 50 | 100 | 1750 | 210 | 193 | 91.9 |
| **SET 8** | 450 | 50 | 39 | 78 | 450 | 50 | 50 | 100 | 450 | 50 | 50 | 100 | 410 | 50 | 50 | 100 | 1760 | 200 | 189 | 94.5 |
| **SET 9** | 450 | 50 | 39 | 78 | 450 | 50 | 50 | 100 | 450 | 50 | 50 | 100 | 410 | 50 | 50 | 100 | 1760 | 200 | 189 | 94.5 |
| **SET 10** | 450 | 50 | 38 | 76 | 450 | 50 | 50 | 100 | 450 | 50 | 50 | 100 | 420 | 40 | 40 | 100 | 1770 | 190 | 178 | 93.7 |
| **TOTAL** | 4500 | 500 | 409 | 81.8 | 4470 | 530 | 517 | 97.5 | 4500 | 500 | 484 | 96.8 | 4140 | 460 | 460 | 100 | 17610 | 1990 | 1870 | **94** |

**Table S6:** Machine learning results for HCC patients.

**SUPPORT VECTOR MACHINE**

|  | **HCC** | | | | **NON-TUMOR TISSUE** | | | | **TOTAL** | | | |
| --- | --- | --- | --- | --- | --- | --- | --- | --- | --- | --- | --- | --- |
|  | **TRAINING** | **TEST** | **CORRECT** | **SENSITIVITY (%)** | **TRAINING** | **TEST** | **CORRECT** | **SPECIFICITY (%)** | **TRAINING** | **TEST** | **CORRECT** | **ACCURACY (%)** |
| **SET 1** | 105 | 12 | 5 | 41.7 | 94 | 11 | 11 | 100 | 199 | 23 | 16 | 69.6 |
| **SET 2** | 106 | 11 | 10 | 90.9 | 94 | 11 | 11 | 100 | 200 | 22 | 21 | 95.5 |
| **SET 3** | 106 | 11 | 11 | 100 | 95 | 10 | 9 | 90 | 201 | 21 | 20 | 95.2 |
| **SET 4** | 105 | 12 | 5 | 41.7 | 93 | 12 | 10 | 83.3 | 198 | 24 | 15 | 62.5 |
| **SET 5** | 105 | 12 | 12 | 100 | 95 | 10 | 10 | 100 | 200 | 22 | 22 | 100 |
| **SET 6** | 104 | 13 | 13 | 100 | 94 | 11 | 10 | 90.9 | 198 | 24 | 23 | 95.8 |
| **SET 7** | 105 | 12 | 11 | 91.7 | 95 | 10 | 10 | 100 | 200 | 22 | 21 | 95.5 |
| **SET 8** | 105 | 12 | 8 | 66.7 | 93 | 12 | 12 | 100 | 198 | 24 | 20 | 83.3 |
| **SET 9** | 105 | 12 | 12 | 100 | 93 | 12 | 12 | 100 | 198 | 24 | 24 | 100 |
| **SET 10** | 107 | 10 | 10 | 100 | 99 | 6 | 5 | 83.3 | 206 | 16 | 15 | 93.8 |
| **TOTAL** | 1053 | 117 | 97 | 82.9 | 945 | 105 | 100 | 95.2 | 1998 | 222 | 197 | **88**.**7** |

**RANDOM FOREST**

|  | **HCC** | | | | **NON-TUMOR TISSUE** | | | | **TOTAL** | | | |
| --- | --- | --- | --- | --- | --- | --- | --- | --- | --- | --- | --- | --- |
|  | **TRAINING** | **TEST** | **CORRECT** | **SENSITIVITY (%)** | **TRAINING** | **TEST** | **CORRECT** | **SPECIFICITY (%)** | **TRAINING** | **TEST** | **CORRECT** | **ACCURACY (%)** |
| **SET 1** | 105 | 12 | 12 | 100 | 94 | 11 | 10 | 90.9 | 199 | 23 | 22 | 95.7 |
| **SET 2** | 106 | 11 | 10 | 90.9 | 94 | 11 | 11 | 100 | 200 | 22 | 21 | 95.5 |
| **SET 3** | 106 | 11 | 11 | 100 | 95 | 10 | 10 | 100 | 201 | 21 | 21 | 100 |
| **SET 4** | 105 | 12 | 12 | 100 | 93 | 12 | 12 | 100 | 198 | 24 | 24 | 100 |
| **SET 5** | 105 | 12 | 12 | 100 | 95 | 10 | 10 | 100 | 200 | 22 | 22 | 100 |
| **SET 6** | 104 | 13 | 13 | 100 | 94 | 11 | 11 | 100 | 198 | 24 | 24 | 100 |
| **SET 7** | 105 | 12 | 12 | 100 | 95 | 10 | 10 | 100 | 200 | 22 | 22 | 100 |
| **SET 8** | 105 | 12 | 11 | 91.7 | 93 | 12 | 12 | 100 | 198 | 24 | 23 | 95.8 |
| **SET 9** | 105 | 12 | 12 | 100 | 93 | 12 | 12 | 100 | 198 | 24 | 24 | 100 |
| **SET 10** | 107 | 10 | 9 | 90 | 99 | 6 | 6 | 100 | 206 | 16 | 15 | 93.8 |
| **TOTAL** | 1053 | 117 | 114 | 97.4 | 945 | 105 | 104 | 99.0 | 1998 | 222 | 218 | **98**.**2** |

**Table S7:** Machine learning results for MFCCC patients.

**SUPPORT VECTOR MACHINE**

|  | **MFCCC** | | | | **NON-TUMOR TISSUE** | | | | **TOTAL** | | | |
| --- | --- | --- | --- | --- | --- | --- | --- | --- | --- | --- | --- | --- |
|  | **TRAINING** | **TEST** | **CORRECT** | **SENSITIVITY (%)** | **TRAINING** | **TEST** | **CORRECT** | **SPECIFICITY (%)** | **TRAINING** | **TEST** | **CORRECT** | **ACCURACY (%)** |
| **SET 1** | 44 | 6 | 5 | 83.3 | 41 | 5 | 5 | 100 | 85 | 11 | 10 | 90.9 |
| **SET 2** | 41 | 5 | 5 | 100 | 45 | 5 | 5 | 100 | 86 | 10 | 10 | 100 |
| **SET 3** | 44 | 6 | 6 | 100 | 40 | 6 | 6 | 100 | 84 | 12 | 12 | 100 |
| **SET 4** | 44 | 6 | 6 | 100 | 41 | 5 | 5 | 100 | 85 | 11 | 11 | 100 |
| **SET 5** | 44 | 6 | 6 | 100 | 40 | 6 | 6 | 100 | 84 | 12 | 12 | 100 |
| **SET 6** | 44 | 6 | 6 | 100 | 41 | 5 | 5 | 100 | 85 | 11 | 11 | 100 |
| **SET 7** | 44 | 6 | 6 | 100 | 40 | 6 | 6 | 100 | 84 | 12 | 12 | 100 |
| **SET 8** | 45 | 5 | 5 | 100 | 41 | 5 | 5 | 100 | 86 | 10 | 10 | 100 |
| **SET 9** | 46 | 4 | 4 | 100 | 43 | 3 | 3 | 100 | 89 | 7 | 7 | 100 |
| **TOTAL** | 396 | 50 | 49 | 98 | 372 | 46 | 46 | 100 | 768 | 96 | 95 | **99** |

**RANDOM FOREST**

|  | **MFCCC** | | | | **NON-TUMOR TISSUE** | | | | **TOTAL** | | | |
| --- | --- | --- | --- | --- | --- | --- | --- | --- | --- | --- | --- | --- |
|  | **TRAINING** | **TEST** | **CORRECT** | **SENSITIVITY (%)** | **TRAINING** | **TEST** | **CORRECT** | **SPECIFICITY (%)** | **TRAINING** | **TEST** | **CORRECT** | **ACCURACY (%)** |
| **SET 1** | 44 | 6 | 6 | 100 | 41 | 5 | 5 | 100 | 85 | 11 | 11 | 100 |
| **SET 2** | 41 | 5 | 5 | 100 | 45 | 5 | 5 | 100 | 86 | 10 | 10 | 100 |
| **SET 3** | 44 | 6 | 6 | 100 | 40 | 6 | 5 | 83.3 | 84 | 12 | 11 | 91.7 |
| **SET 4** | 44 | 6 | 6 | 100 | 41 | 5 | 5 | 100 | 85 | 11 | 11 | 100 |
| **SET 5** | 44 | 6 | 6 | 100 | 40 | 6 | 6 | 100 | 84 | 12 | 12 | 100 |
| **SET 6** | 44 | 6 | 6 | 100 | 41 | 5 | 5 | 100 | 85 | 11 | 11 | 100 |
| **SET 7** | 44 | 6 | 6 | 100 | 40 | 6 | 6 | 100 | 84 | 12 | 12 | 100 |
| **SET 8** | 45 | 5 | 5 | 100 | 41 | 5 | 5 | 100 | 86 | 10 | 10 | 100 |
| **SET 9** | 46 | 4 | 4 | 100 | 43 | 3 | 3 | 100 | 89 | 7 | 7 | 100 |
| **TOTAL** | 396 | 50 | 50 | 100 | 372 | 46 | 45 | 97.8 | 768 | 96 | 95 | **99** |

**Table S8:** Machine learning results for HCC and MFCCC patients.

**SUPPORT VECTOR MACHINE**

|  | **TUMOR** | | | | | | | | **NON-TUMOR TISSUE** | | | | | | | | **TOTAL** | | | |
| --- | --- | --- | --- | --- | --- | --- | --- | --- | --- | --- | --- | --- | --- | --- | --- | --- | --- | --- | --- | --- |
|  | **HCC** | | | | **MFCCC** | | | | **HCC** | | | | **MFCCC** | | | |  | | | |
|  | **TR** | **TE** | **COR** | **SENS. (%)** | **TR** | **TE** | **COR** | **SENS. (%)** | **TR** | **TEST** | **COR** | **SPEC. (%)** | **TR** | **TE** | **COR** | **SPEC. (%)** | **TR** | **TE** | **C** | **ACC (%)** |
| **SET 1** | 45 | 5 | 5 | 100 | 45 | 5 | 5 | 100 | 45 | 5 | 5 | 100 | 41 | 5 | 5 | 100 | 176 | 20 | 20 | 100 |
| **SET 2** | 45 | 5 | 5 | 100 | 45 | 5 | 5 | 100 | 45 | 5 | 5 | 100 | 42 | 4 | 4 | 100 | 177 | 19 | 19 | 100 |
| **SET 3** | 45 | 5 | 5 | 100 | 45 | 5 | 5 | 100 | 45 | 5 | 5 | 100 | 41 | 5 | 5 | 100 | 176 | 20 | 20 | 100 |
| **SET 4** | 45 | 5 | 5 | 100 | 43 | 4 | 4 | 100 | 45 | 5 | 5 | 100 | 42 | 4 | 4 | 100 | 175 | 18 | 18 | 100 |
| **SET 5** | 45 | 5 | 5 | 100 | 44 | 6 | 6 | 100 | 45 | 5 | 5 | 100 | 42 | 4 | 4 | 100 | 176 | 20 | 20 | 100 |
| **SET 6** | 45 | 5 | 4 | 80 | 46 | 4 | 4 | 100 | 45 | 5 | 5 | 100 | 41 | 5 | 5 | 100 | 177 | 19 | 18 | 94.7 |
| **SET 7** | 45 | 5 | 4 | 80 | 44 | 6 | 6 | 100 | 45 | 5 | 4 | 80 | 41 | 5 | 5 | 100 | 175 | 21 | 19 | 90.5 |
| **SET 8** | 45 | 5 | 5 | 100 | 45 | 5 | 5 | 100 | 45 | 5 | 5 | 100 | 41 | 5 | 5 | 100 | 176 | 20 | 20 | 100 |
| **SET 9** | 45 | 5 | 5 | 100 | 45 | 5 | 5 | 100 | 45 | 5 | 5 | 100 | 41 | 5 | 5 | 100 | 176 | 20 | 20 | 100 |
| **SET 10** | 45 | 5 | 5 | 100 | 45 | 5 | 5 | 100 | 45 | 5 | 5 | 100 | 42 | 4 | 4 | 100 | 177 | 19 | 19 | 100 |
| **TOTAL** | 450 | 50 | 48 | 96 | 447 | 50 | 50 | 100 | 450 | 50 | 49 | 98 | 414 | 46 | 46 | 100 | 1761 | 196 | 193 | **98**.**5** |

**RANDOM FOREST**

|  | **TUMOR** | | | | | | | | **NON-TUMOR TISSUE** | | | | | | | | **TOTAL** | | | |
| --- | --- | --- | --- | --- | --- | --- | --- | --- | --- | --- | --- | --- | --- | --- | --- | --- | --- | --- | --- | --- |
|  | **HCC** | | | | **MFCCC** | | | | **HCC** | | | | **MFCCC** | | | |  | | | |
|  | **TR** | **TE** | **COR** | **SENS. (%)** | **TR** | **TE** | **COR** | **SENS. (%)** | **TR** | **TEST** | **COR** | **SPEC. (%)** | **TR** | **TE** | **COR** | **SPEC. (%)** | **TR** | **TE** | **C** | **ACC (%)** |
| **SET 1** | 45 | 5 | 5 | 100 | 45 | 5 | 5 | 100 | 45 | 5 | 4 | 80 | 41 | 5 | 5 | 100 | 176 | 20 | 19 | 95 |
| **SET 2** | 45 | 5 | 3 | 60 | 45 | 5 | 5 | 100 | 45 | 5 | 5 | 100 | 42 | 4 | 4 | 100 | 177 | 19 | 17 | 89.5 |
| **SET 3** | 45 | 5 | 5 | 100 | 45 | 5 | 5 | 100 | 45 | 5 | 4 | 80 | 41 | 5 | 5 | 100 | 176 | 20 | 19 | 95 |
| **SET 4** | 45 | 5 | 5 | 100 | 43 | 4 | 4 | 100 | 45 | 5 | 5 | 100 | 42 | 4 | 4 | 100 | 175 | 18 | 18 | 100 |
| **SET 5** | 45 | 5 | 5 | 100 | 44 | 6 | 6 | 100 | 45 | 5 | 5 | 100 | 42 | 4 | 4 | 100 | 176 | 20 | 20 | 100 |
| **SET 6** | 45 | 5 | 4 | 80 | 46 | 4 | 3 | 75 | 45 | 5 | 5 | 100 | 41 | 5 | 5 | 100 | 177 | 19 | 17 | 89.5 |
| **SET 7** | 45 | 5 | 3 | 60 | 44 | 6 | 6 | 100 | 45 | 5 | 5 | 100 | 41 | 5 | 5 | 100 | 175 | 21 | 19 | 90.5 |
| **SET 8** | 45 | 5 | 4 | 80 | 45 | 5 | 5 | 100 | 45 | 5 | 5 | 100 | 41 | 5 | 5 | 100 | 176 | 20 | 19 | 95 |
| **SET 9** | 45 | 5 | 5 | 100 | 45 | 5 | 5 | 100 | 45 | 5 | 5 | 100 | 41 | 5 | 5 | 100 | 176 | 20 | 20 | 100 |
| **SET 10** | 45 | 5 | 4 | 80 | 45 | 5 | 5 | 100 | 45 | 5 | 5 | 100 | 42 | 4 | 4 | 100 | 177 | 19 | 18 | 94.7 |
| **TOTAL** | 450 | 50 | 43 | 86 | 447 | 50 | 49 | 98 | 450 | 50 | 48 | 96 | 414 | 46 | 46 | 100 | 1761 | 196 | 186 | **94.9** |
